# Special Issue “Bioinformatics of Gene Regulations and Structure–2025”

**DOI:** 10.3390/ijms27188334

**Published:** 2026-09-19

**Authors:** Yuriy L. Orlov, Anastasia A. Anashkina, Inessa A. Minenko, Nikolay A. Kolchanov

**Affiliations:** 1 Center of Digital Health and AI in Medicine, I.M. Sechenov First Moscow State Medical University of the Ministry of Health of the Russian Federation (Sechenov University), 119991 Moscow, Russia; 2 Agrarian and Technological Institute, Peoples’ Friendship University of Russia, 117198 Moscow, Russia; 3 Department of Mathematics, Novosibirsk State University, 630090 Novosibirsk, Russia; 4 Engelhardt Institute of Molecular Biology, Russian Academy of Sciences, 119991 Moscow, Russia; anastasia.a.anashkina@mail.ru; 5 Departmentof Sport Medicine and Medical Rehabilitation, I.M. Sechenov First Moscow State Medical University of the Ministry of Health of the Russian Federation (Sechenov University), 119991 Moscow, Russia; minenko_i_a@staff.sechenov.ru; 6 Institute of Cytology and Genetics, Siberian Branch of the Russian Academy of Sciences, 630090 Novosibirsk, Russia; kol@bionet.nsc.ru

This article overviews current trends in the molecular mechanisms of gene expression regulation studies published in the “Bioinformatics of Gene Regulations and Structure—2025” Special Issue (https://www.mdpi.com/journal/ijms/special_issues/3I56FG1O3A, accessed on 30 July 2026). This topical issue collects papers on genetics and bioinformatics, on the topic of gene expression regulation, from the Institute of Cytology and Genetics of the Siberian Branch of the Russian Academy of Sciences (ICG SB RAS) in Novosibirsk (https://www.icgbio.ru/bgrs2026, accessed on 30 July 2026), discussed at the “Bioinformatics of Genome Regulation and Structure/Systems Biology” (BGRS/SB) conference series last year and related systems biology meetings in Russia.

We have already organized several successful Special Issues on the bioinformatics of gene expression, including “Molecular Mechanisms of Gene Expression: “Bioinformatics of Gene Regulations and Structure”” (https://www.mdpi.com/journal/ijms/special_issues/Bioinformatics_Genomics, accessed on 30 July 2026) and “New Sights into Bioinformatics of Gene Regulations and Structure” (https://www.mdpi.com/journal/ijms/special_issues/MVA479KFR7, accessed on 30 July 2026), and then a series of topic issues on the medical applications of the bioinformatics of gene expression: “25 Anniversary of Bioinformatics of Genome Regulation and Structure Conference Series” (https://www.mdpi.com/journal/ijms/special_issues/0LGA6103S5, accessed on 30 July 2026) [1,2,3]. The central problems include the analysis of the molecular mechanisms of gene expression regulation, the analysis of transcription regulation by protein transcription factors, regulatory gene network interaction analysis, and applications of modern AI tools in bioinformatics. This Special Issue is focused on the bioinformatics of gene expression, as before [2,3]. The topics of interest include the analysis of gene expression regulation, applications of bioinformatics to 3D genomics technologies, interaction network analysis, and computational genomics for model organisms.

The BGRS conference series started in 1998, presenting unique genetics and genomics discoveries biannually till recent years. To commemorate the history of BGRS in Novosibirsk, we note the Belyaev conference on genetics and evolution “Belyaev Readings—2017” (http://conf.bionet.nsc.ru/belyaev100/en, accessed on 30 July 2026) devoted to the memory of Academician Dmitry K. Belyaev (1917–1985), the founder of the Institute of Cytology and Genetics [4]. His research on the genetics of the behavior and the domestication of animals [5,6] laid the background for modern neurobiology studies continued in this issue. The research areas of gene expression and genome regulation discussed at the BGRS series [1,2] include sequence analysis, transcription regulation, gene network modeling and medical applications of gene function analysis [7,8].

We open this collection of papers with theoretical work on gene expression (Contributions 1 and 2). Pavel Kondrakhin and Fedor Kolpakov (Contribution 1) (https://doi.org/10.3390/ijms27010490) presented a modular mathematical model of neuronal activity, designed to simulate the dynamics of the main molecular targets of antiepileptic drugs and their pharmacological effects. The model was developed based on several existing synaptic transmission models that capture cellular processes crucial to the pathology of epilepsy [9,10].

Erik Tadevosyan and co-authors (Contribution 2) (https://doi.org/10.3390/ijms262411977) discussed age-related dependencies in gene expression. This fundamental study used single-cell RNA-seq data and scGPT, a large transcriptomic model, to predict chronological age groups [7,11,12]. The authors demonstrated that scGPT does capture age-related dependencies in single-cell data and can be utilized to discover novel candidate gene perturbations—potential targets to be validated as anti-aging interventions.

The next block of papers considers applications in model organisms. Natalya Bondar and colleagues (Contribution 3) (https://doi.org/10.3390/ijms27010018) used a mouse model to study the molecular mechanisms of social stress, constituting a challenge for neurobiology [13]. The authors investigated the epigenetic signatures (H3K4me3) of social defeat stress in the prefrontal cortex of C57BL/6 mice, continuing model research on laboratory animals [14,15]. The analysis of differential H3K4me3 modifications in gene promoter regions in the mouse stress groups revealed genes encoding transcription, as well as postsynaptic density, proteins (Shank2, Shank1, and Gria2) associated with stress sensitivity and the onset of depression.

Valentina Grushina and colleagues (Contribution 4) (https://doi.org/10.3390/ijms262311407) analyzed gene expression in a model organism—chickens [16]. Gene expression from promoters is influenced by interactions with genomic enhancers located within the same topologically associating domain (TAD) [17]. The authors examined the correlation between gene and enhancer activities within individual TADs across multiple tissues in slow- and fast-growing chickens. It was shown that enhancer-mediated regulation appears to activate key pathways involved in transcriptional control and nucleic acid biosynthesis.

The current understanding of avian enhancers remains less developed compared to that of mammals [16,18]. The next study by the same group of authors, Grushina et al. (Contribution 5) (https://doi.org/10.3390/ijms262210986), utilized CAGE-seq data from chicken tissues obtained through the “Genetic Technologies in Poultry” project to predict enhancers in the chicken genome. The analysis revealed that the proportion of enhancer-associated DNA in chickens is quite similar to that observed in mammals, encompassing about 9% of the entire chicken genome, similar to the estimate made by the ChickenGTEx project [19]. A group of enhancers was significantly expressed among the tissues studied, highlighting gene expression regulation in chickens [20].

Finally, we note medical applications presented in this Special Issue. Yebin Son and Jae Yong Ryu (Contribution 6) (https://doi.org/10.3390/ijms26199530) investigated anticancer targets in ovarian cancer using genomic drug sensitivity data. PARP inhibitors exploit synthetic lethality in BRCA1/2-mutated ovarian cancers but are limited by emerging therapeutic resistance [21]. The authors investigated biomarkers associated with PARP inhibitor responses. Drug sensitivity analysis revealed that BRCA1, MLL2, NF1, and SMARCA4 mutations enhance sensitivity to PARP inhibitors in ovarian cancer.

The study by Zeb Hussain et al. (Contribution 7) (https://doi.org/10.3390/ijms27125391) presents a phylogenetic analysis of microbiology data from an Intensive Care Unit. The global dissemination of carbapenem resistance is predominantly facilitated by plasmid-mediated carbapenemase genes, notably blaOXA-48-like genes [22,23]. Understanding their evolutionary relationships is essential for monitoring their spread and informing therapeutic strategies. The study investigated the phylogenetic relationships and structural conservation of blaOXA-48-like carbapenemase genes in multiple Gram-negative bacterial species [23]. The authors found that blaOXA-48-like carbapenemases have high evolutionary stability.

Andrey R. Karpenko and colleagues (Contribution 8) (https://doi.org/10.3390/ijms26188967) studied a medical case of severe COVID-19 [24]. Human heat shock proteins (HSPs) play a dual role by either protecting host cells or acting to advance viral spread in viral infections [25]. Genetic variants of human heat shock proteins raised interest in the context of severe COVID-19 risk [26,27]. In this study, 1228 subjects were genotyped for 20 SNPs in genes encoding HSPs and their regulatory regions. Several SNPs were found. The study provides preliminary evidence that SNPs of heat shock proteins can significantly modulate the risk of severe COVID-19.

To recap current trends in gene expression studies, we note AI applications in bioinformatics (https://mathai.club/index.html, https://enigma.ist/journals, accessed on 30 July 2026) discussed at the mathematical conference series initiated in Novosibirsk and Moscow (https://congrsysalgbai.ru/, accessed on 30 July 2026). Recent special journal issues on the bioinformatics of gene expression regulation include an issue of *Frontiers in Genetics* (https://www.frontiersin.org/research-topics/40408/bioinformatics-of-genome-regulation-and-systems-biology-volume-iii, https://www.frontiersin.org/research-topics/21036/high-throughput-sequencing-based-investigation-of-chronic-disease-markers-and-mechanisms, accessed on 30 July 2026) and a series published by *BioMedCentral* (https://bmcgenomics.biomedcentral.com/articles/supplements/volume-20-supplement-3, accessed on 30 July 2026) [28].

This year, we continue the series of Special Issues in the *Gene Expression* journal (https://www.xiahepublishing.com/journal/ge/features/bioinformatics, accessed on 30 July 2026). In addition, the *IJMS* Special Issue (https://www.mdpi.com/journal/ijms/special_issues/C0F2W4J5X5, accessed on 30 July 2026) continues the trend set by the earlier journal issues and paper collections on advances in computer genomics and bioinformatics presented at the Young Scientist Schools “Systems biology and bioinformatics” (SBB) – education course series [29].

We hope that readers find these materials to be interesting and stimulating for the gene expression field [30], and we will continue to collect papers on this topic for ongoing issues including plant biology (https://www.mdpi.com/journal/ijms/special_issues/1D646O0T7V, accessed on 30 July 2026).
